# Phenotypic and Genotypic Characterization of Atypical *Listeria monocytogenes* and *Listeria innocua* Isolated from Swine Slaughterhouses and Meat Markets

**DOI:** 10.1155/2014/742032

**Published:** 2014-05-28

**Authors:** Luisa Zanolli Moreno, Renata Paixão, Debora Dirani Sena de Gobbi, Daniele Cristine Raimundo, Thais Sebastiana Porfida Ferreira, Andrea Micke Moreno, Ernesto Hofer, Cristhiane Moura Falavina dos Reis, Glavur Rogério Matté, Maria Helena Matté

**Affiliations:** ^1^Laboratório Prática de Saúde Pública, Faculdade de Saúde Pública, Universidade de São Paulo, Avenida Doutor Arnaldo, No. 715, 01246 904 São Paulo, SP, Brazil; ^2^Laboratório de Sanidade Suína e Virologia, Faculdade de Medicina Veterinária e Zootecnia, Universidade de São Paulo, Avenida Professor Doutor Orlando Marques de Paiva, No. 87, Cidade Universitária, 05508 270 São Paulo, SP, Brazil; ^3^Laboratório de Zoonoses Bacterianas, Fundação Instituto Oswaldo Cruz, Avenida Brasil 4365, Manguinhos, 21040 360 Rio de Janeiro, RJ, Brazil

## Abstract

In the last decade, atypical *Listeria monocytogenes* and *L. innocua* strains have been detected in food and the environment. Because of mutations in the major virulence genes, these strains have different virulence intensities in eukaryotic cells. In this study, we performed phenotypic and genotypic characterization of atypical *L. monocytogenes* and *L. innocua* isolates obtained from swine slaughterhouses and meat markets. Forty strains were studied, including isolates of *L. monocytogenes* and *L. innocua* with low-hemolytic activity. The isolates were characterized using conventional phenotypic *Listeria* identification tests and by the detection and analysis of *L. monocytogenes*-specific genes. Analysis of 16S rRNA was used for the molecular identification of the *Listeria* species. The *L. monocytogenes* isolates were positive for all of the virulence genes studied. The atypical *L. innocua* strains were positive for *hly, plcA,* and *inlC*. Mutations in the InlC, InlB, InlA, PI-PLC, PC-PLC, and PrfA proteins were detected in the atypical isolates. Further *in vitro* and transcriptomic studies are being developed to confirm the role of these mutations in *Listeria* virulence.

## 1. Introduction

*Listeria monocytogenes* and* L. innocua* are closely related species of the Gram-positive genus* Listeria*. They are widely distributed in the environment and frequently isolated from food.* L. monocytogenes* is the causative agent of listeriosis, a foodborne disease with a high fatality rate (20–30%) that mostly affects the elderly, neonates, and immunocompromised individuals [1, 2].* L. monocytogenes* cannot be distinguished from other* Listeria* species using conventional isolation methods. Standard biochemical methods and selective and differential media are used for the identification of* L. monocytogenes *[3, 4]; however, some* L. ivanovii*,* L. innocua,* and* L. seeligeri* strains generate similar results to* L. monocytogenes *in these tests [5–7]. Therefore, it is necessary to confirm the virulence characteristics of* L. monocytogenes* to distinguish the* Listeria* species.

The best-characterized* L. monocytogenes* virulence factors are listeriolysin O (LLO), phosphatidylinositol phospholipase C (PI-PLC), and the internalins A and B (InlA and InlB). LLO and PI-PLC are encoded by the* hly* and* plcA* genes, respectively, which belong to the virulence gene cluster* Listeria* pathogenicity island 1 (LIPI-1), which contains the major virulence genes of* L. monocytogenes* [8]. Few atypical* L. innocua* strains have been reported to contain* L. monocytogenes*-specific genes and exhibit phenotypic characteristics similar to* L. monocytogenes* such as weak hemolysis [6, 7, 9]. Furthermore, certain low-hemolytic* L. monocytogenes* strains retain their virulence despite the presence of mutations in major virulence genes [10–12]. The existence of these atypical strains indicates that traditional phenotypic and genotypic characterization methods must be used with care and that further studies are required to improve the identification of* Listeria* isolates.

This study used phenotypic and genotypic methods to characterize atypical* L. monocytogenes* and* L. innocua* isolates obtained from swine slaughterhouses and meat markets in Sao Paulo State, Brazil.

## 2. Material and Methods 

### 2.1. Bacterial Strains and Culture Conditions

Forty* Listeria* sp. isolates were studied. Of these, 25 were isolated from pork, slaughterhouses, and markets (15 isolates of* L. monocytogenes* and 10 of* L. innocua*), 11 isolates of* L. monocytogenes* were obtained from human infections, and four were control strains (*L. monocytogenes* ATCC 19115 and ATCC 19111 and* L. innocua* ATCC 33090 and CLIP 12612) (Table 1). The environmental and pork isolates were isolated as described by Moreno et al. [13]; the clinical strains and* Listeria* controls were obtained from the Public Health Laboratory (School of Public Health, University of Sao Paulo) and Laboratory of Swine Health (School of Veterinary Medicine and Animal Science, University of Sao Paulo) collections. The environmental and pork isolates were obtained from different swab samples taken from the slaughterhouses environment and carcasses from Sao Paulo State; the clinical isolates were obtained from the blood, placenta, and cerebrospinal fluid samples of different patients from different Brazilian states (Tables 1 and 2).

The isolates were maintained in a stock medium containing glycerol at −80°C. The isolates were reactivated in brain-heart infusion (BHI) medium (Difco, Sparks, MD, USA) and plated on tryptone soy agar supplemented with yeast (TSAYE) (Oxoid, Lenexa, USA) to isolate pure colonies before use.

### 2.2. Conventional Listeria Identification Tests

The isolates were serotyped using polyclonal antisera produced against Listeria somatic and flagellar antigens in rabbits, according to the method described by Seeliger and Höhne [14]. The isolates were also characterized by catalase, motility, and biochemical tests including acid production from D-xylose, D-mannitol, L-rhamnose, and *α*-methyl-D-mannoside. Cultivation in selective agar* Listeria* according to Ottaviani and Agosti (ALOA) (Biolife, Milan, Italy) was used to identify* L. monocytogenes* isolates, and *β*-hemolysis was assessed by sting inoculation on 5% sheep blood agar.

### 2.3. Detection of* L. monocytogenes*-Specific Genes

Genomic DNA extraction was performed as described by Ausubel et al. [15]. All isolates were screened for the* inlA*,* inlB*,* inlC*,* inlJ*,* hly*,* prfA*,* plcA,* and* plcB* genes. The primers described by Johnson et al. [6], Liu et al. [16], and Jung et al. [17] were used for detection of* prfA*,* inlC *and* inlJ, *and* inlA*, respectively. Specific primers were designed for the complete amplification of the virulence genes (Table 3). The PCRs were performed using an* Eppendorf Mastercycler gradient* thermal cycler. Each reaction (25 *μ*L) contained 5 *μ*L of genomic DNA, MilliQ water, 10X PCR buffer, 1.5 mM MgCl_2,_ 200 *μ*M of dNTPs (Fermentas, Burlington, Canada), 200 *μ*M of each primer, and 1.25 U of Taq DNA polymerase (Promega). The PCR programs were as follows: 30 cycles of denaturation at 94°C for 1 min, annealing at primer-specific temperature for 1–1.5 min, elongation at 72°C for 1 min per 1 Kb, and final extension at 72°C for 10 min. The amplified products were separated by electrophoresis on 1.5% agarose gels and stained with ethidium bromide (1 *μ*g/mL). The molecular weights of the products were determined using the 1 Kb Plus DNA Ladder (Fermentas, Burlington, Canada).

### 2.4. DNA Sequencing

The amplified fragments were purified using the Illustra GFX PCR DNA and Gel Band Purification kit (*GE Healthcare*) according to the manufacturer's protocol and sequenced directly at Genomic (Genomic Engenharia Molecular, Sao Paulo, Brazil). DNA sequencing was performed on an Applied Biosystems 3130xl DNA analyzer using the BigDye Terminator v3.1 cycle sequencing kit.

### 2.5. Detection of Mutations in* L. monocytogenes* Virulence Genes

Sequence analysis was performed using the BIOEDIT Sequence Alignment Editor 7.0.9 [18]. The obtained sequences of the virulence genes were compared to previously published* L. monocytogenes* sequence accessions from GenBank (NCBI, Bethesda, USA). The sequencing products were edited and compared with the sequences available in the GenBank database by manual alignment and using the ClustalW application. The nucleotide sequences obtained were translated into their corresponding amino acid sequences by the Nucleotide Translate application. Subsequently, the amino acid sequences were analyzed to identify changes in the compositions of their respective proteins, which might modify or eliminate protein functions.

### 2.6. Identification of Protein Domains and Prediction of Secondary Structures

The domains of InlC, InlB, InlA, PI-PLC, PC-PLC, and Hly from reference strain* L. monocytogenes* EGD-e were determined using the PROSITE database [19] of the ExPASy server (SIB, Swiss Institute of Bioinformatics). The locations of these domains were compared to the mutations identified in the studied isolates.

### 2.7. Species-Level Identification by 16S rRNA Amplification and Phylogenetic Analysis

Species identity was confirmed using 16S rRNA analysis. The primers and amplification protocol described by Thompson et al. [20] were used to amplify complete 16S rRNA genes. The fragments were sequenced and phylogenetic analysis was performed using the Mega 5.10 software [21]. The dendrogram was constructed using the maximum-likelihood method with the Tamura-3-parameter model.

### 2.8. Nucleotide Sequence Accession Numbers

All DNA sequences from this study were deposited in GenBank under the accession numbers KC618415-KC618420, KC666995-KC667019, KC808518-KC808549, and KC808567-KC808583.

## 3. Results

### 3.1. Conventional Listeria Identification Tests

The phenotypic characterization of* Listeria* sp. isolates is shown in Tables 1 and 2. Five atypical* L. innocua* isolates (*Lin5–9*) and six low-hemolytic* L. monocytogenes* (*Lm4*,* Lm33,* and* Lm28–31*) isolates were observed. The atypical* L. innocua* isolates exhibited phenotypic characteristics similar to* L. monocytogenes* with weak hemolysis and subtle halo in ALOA cultivation. These isolates could be distinguished only by serotyping, which revealed that the atypical isolates were* L. innocua* serotype 6a.

### 3.2. Detection and Analysis of* L. monocytogenes* Virulence Genes

The detection and complete amplification of the* inlB*,* inlC*,* plcA*,* plcB*,* hly,* and* prfA *genes were performed using previously published primers and primers designed in this study. The* inlA* and* inlJ* genes were only partially amplified using the primers* inlA In-Fw*/*inlA Detec-Rv, *designed in this study,and* inlJ-Fw*/*inlJ-Rv*, which were described by Liu et al. [16]. All* L. monocytogenes* isolates including the six low-hemolytic isolates (*Lm4*,* Lm33, *and* Lm28–31*) contained the studied genes. The five atypical* L. innocua* isolates (*Lin5–9*) contained* inlC* and* plcA* and fragments of the* hly *gene (Table 4).

**Figure 1:**
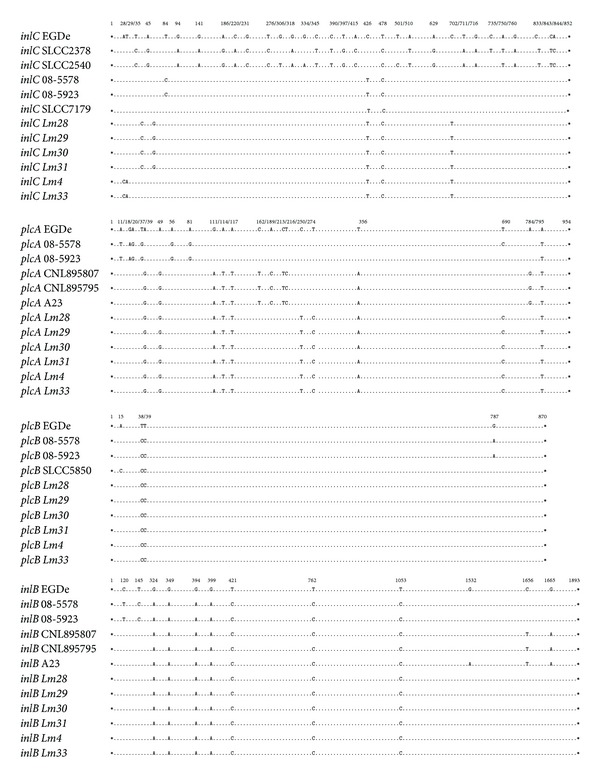
Nucleotide substitutions detected in the* inlC*,* plcA*,* plcB,* and* inlB* genes. The* Lm28*–*31*,* Lm4*, and* Lm33* isolates were aligned with* L. monocytogenes* EGDe and the previously described mutant strains. Asterisks indicate the start and stop codons, dots represent identical nucleotides, and numbers indicate the positions of the substitutions.

**Figure 2:**
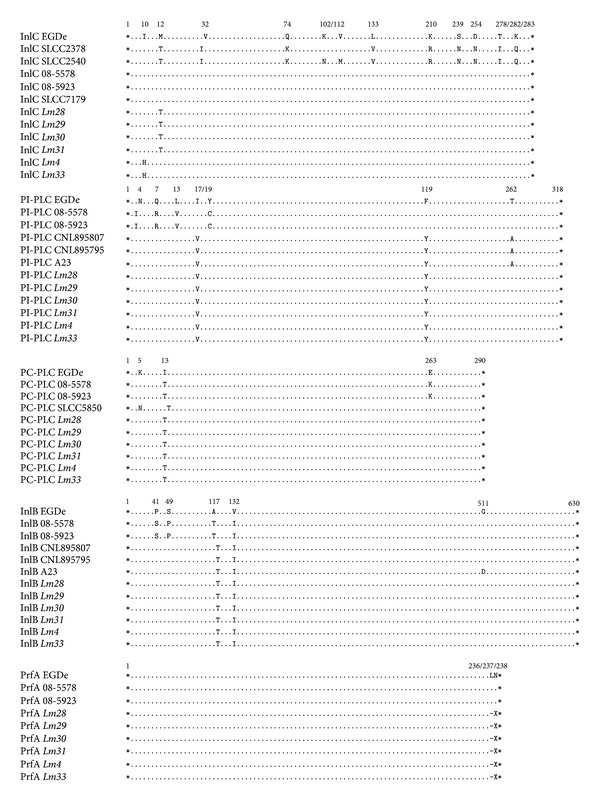
Amino acids substitutions in the InlC, PI-PLC, PC-PLC, InlB, and PrfA proteins. The* Lm28*–*31*,* Lm4*, and* Lm33* isolates were aligned with* L. monocytogenes* EGDe and the previously described mutant strains. Asterisks indicate the start and stop codons, dots represent identical amino acids, and numbers indicate the positions of the substitutions.

Nucleotide substitutions were detected in* inlC*,* inlB*,* inlA*,* plcA*,* plcB*, and* prfA*, only in the six low-hemolytic* L. monocytogenes* isolates (*Lm4*,* Lm33*, and* Lm28–31*). Seven substitutions were detected in the* inlC* gene; however, only the transition of adenine to cytosine and the inversion of thiamine to adenine at codon 10 led to the mutation Ile10His, and the transition of thiamine to cytosine at codon 12 resulted in the mutation Met12Thr. Ten substitutions were detected in* plcA*, leading to the mutations Ile17Val and Phe119Tyr in the PI-PLC. In the* plcB* sequence, only two transitions of thiamine to cytosine were identified at codon 13, which resulted in the mutation Ile13Thr. Seven substitutions were detected in* inlB*; however, only the transitions of adenine to guanine at codons 117 and 132 resulted in the mutations Ala117Thr and Val132Ile (Figures 1 and 2).

**Figure 3:**
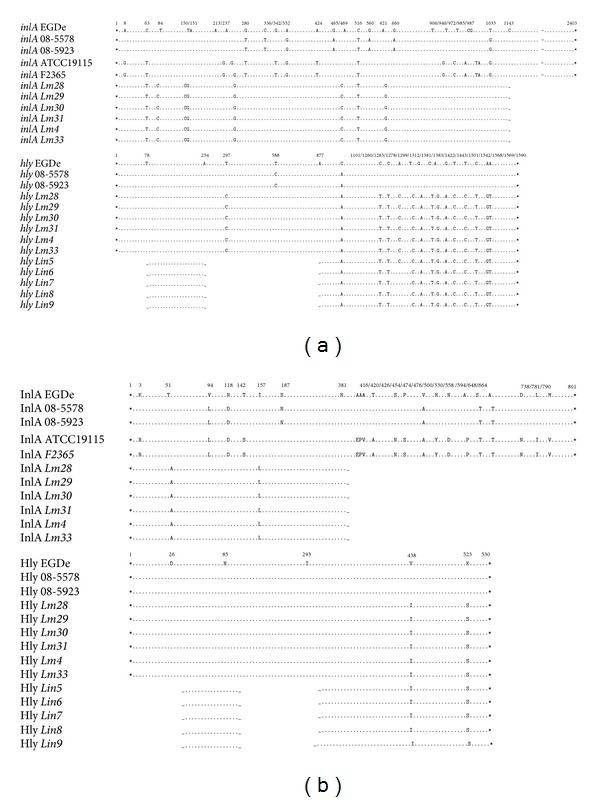
Nucleotide substitutions detected in* inlA* and* hly* (a) and mutations identified in InlA and Hly (b). The* Lm28*–*31*,* Lm4*,* Lm33*, and* Lin5–9* isolates were aligned with* L. monocytogenes* EGDe and previously described mutant strains. Asterisks indicate the start and stop codons, dots represent identical amino acids, and numbers indicate the positions of the substitutions. Gaps represent the regions that were not amplified.

A deletion of five nucleotides was also detected in the* prfA* sequence, leading to the deletion of codons 236 and 237 in the* Lm4*,* Lm33*, and* Lm28–31* isolates. Eight substitutions were detected in the* inlA* fragments of the low-hemolytic* L. monocytogenes* isolates, resulting in the mutations Thr51Ala and Ile157Leu (Figure 3). The* Lm4*,* Lm33*, and* Lm28–31* isolates also contained 15 substitutions in the* hly* sequence, whereas the* Lin5 *and* Lin6–9 *isolates only contained 14 and 13 of these substitutions, respectively. However, all these atypical isolates contained only the mutations Val438Ile and Lys523Ser (Figure 3).

### 3.3. Identification of Protein Domains

Of the identified mutations, only Ala117Thr and Val132Ile in InlB and Ile157Leu in InlA were located in the leucine-rich repeat (LRR) domains that are characteristic of these proteins. The Phe119Tyr mutation in PI-PLC was also located in the PI-PLC X-box domain. The thiol-activated cytolysin signature motifs in Hly and the zinc-dependent phospholipase C domain in PC-PLC presented distinct locations of the mutations identified in the respective proteins.

### 3.4. Species Confirmation by 16S rRNA Phylogenetic Analysis

**Figure 4:**
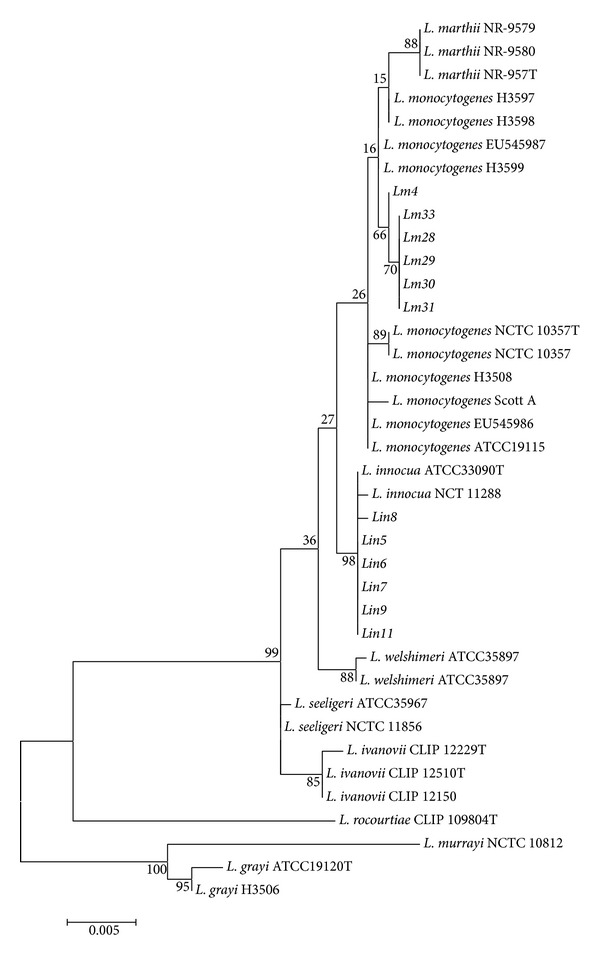
Dendrogram showing the evolutionary relationships among the* Listeria* isolates based on the 16S rRNA nucleotide sequences. The dendrogram was constructed using the maximum-likelihood method (Tamura-3-parameter model) with the MEGA 5.10 software. The bootstrap values presented at corresponding branches were evaluated using 500 replicates.

From the amplification and analysis of the 16S rRNA genes, a dendrogram was constructed, which allowed the distinction of* L. monocytogenes* and* L. innocua* species. The dendrogram contained three major groups; the first group consisted of* L. grayi* and* L. murrayi*, the second group contained* L. rocourtiae*, and the third group consisted of clusters of* L. monocytogenes* and* L. marthii*,* L. innocua*,* L. welshimeri*,* L. seeligeri*, and* L. ivanovii* (Figure 4). The isolates* Lin5* ***–*** *9* and* Lin11* were grouped with the standard strains of* L. innocua*, whereas the isolates* Lm28–31*,* Lm4*, and* Lm33* were grouped together with the standard strains of* L. monocytogenes*.

## 4. Discussion

Studies on* Listeria* virulence mechanisms have become important in recent decades because this microorganism is used as a model of intracellular infection.* L. monocytogenes* virulence factors have been described, and their mechanisms of action and respective genes have been studied using distinct molecular techniques and* in vivo* and* in vitro* experiments. In addition to the use of* Listeria* as a model organism, there is great interest in studying this organism because of the increasing incidence of listeriosis in the United States of America (USA) and Europe [23, 24].

Our results using conventional* Listeria* identification tests are consistent with the subjectivity and ambiguity of phenotypic tests that have been discussed in the last decade [6, 7]. Although these conventional methods are still utilized, biochemical and phenotypic tests yield variable results during the identification of* Listeria* species and serotypes, and the emergence of atypical isolates has further increased the uncertainty of the application of these tests. From a public health perspective, a drastic measure could be adopted to classify all isolates with doubtful hemolytic status as* L. monocytogenes* or as isolates with pathogenic risk without major efforts to identify the species and serovars. However, for better epidemiological, microbiological, and evolutionary understanding, it is important to identify and characterize the phenotypes and molecular features of these atypical isolates.

This study aimed to detect the* hly*,* plcA*,* plcB*,* prfA*,* inlA*,* inlB*,* inlC*, and* inlJ* genes in* L. monocytogenes* and* L. innocua* isolates. These genes are characteristic of* L. monocytogenes* and are essential for intracellular infection. The presence of these genes in isolates from meat and the environment suggests the pathogenic potential of these isolates and a risk to human health. We detected these virulence genes in all* L. monocytogenes* isolates including the six low-hemolytic isolates (*Lm4*,* Lm33*, and* Lm28–31*); additionally, the five atypical* L. innocua* isolates (*Lin5–9*) contained the* inlC*,* hly*, and* plcA* genes.

Our results are consistent with the data of Johnson et al. [6] and Volokhov et al. [7], who identified some* L. monocytogenes *virulence genes in* L. innocua* strains with atypical hemolysis. Therefore, the use of traditional PCR methods based mostly on the detection of* hly* and* plcA* for the distinction of* Listeria* pathogenic species should be reconsidered because these methods do not enable the distinction of atypical isolates. Accurate identification of* Listeria* species was possible only by the complete sequencing and phylogenetic analysis of the 16S rRNA gene (Figure 4). We propose that the detection of* prfA*,* plcB*, and* inlB *might be a better and reliable alternative to enable the rapid distinction of* L. monocytogenes *and* L. innocua*. We also suggest that analysis of the complete 16S rRNA gene sequences is important for the accurate identification of* Listeria* species.

The* inlC *and* plcA* genes from the atypical* L. innocua* isolates did not contain nucleotide substitutions and mutations in their respective proteins. The only mutations identified in these isolates were the Val438Ile and Lys523Ser in Hly. The* hly* gene could not be completely amplified, but this might be due to insertions or deletions between the detected fragments. However, the hemolytic phenotypes of these atypical isolates suggest that despite the difficulty in amplifying this locus there were no gross alterations in Hly function. Further studies will be carried out to confirm and quantify* hly* expression.

Because the atypical* L. innocua* isolates presented the low-hemolytic phenotype and halo in ALOA cultivation, we concluded that these isolates produce at least Hly and PI-PLC. Since the only detected mutations were not located in the thiol-activated cytolysin signature motifs in Hly, the low expression of the* hly* and* plcA* genes might be due to altered promoter activity. As the* prfA* gene was also not detected in these isolates, we suggest that a secondary promoter might activate the expression of* hly* and* plcA* and originate the observed phenotype. However, further* in vitro* and proteomic studies are necessary to verify the activity and integrity of these virulence factors.

The mutations detected in InlB and PI-PLC in the low-hemolytic* L. monocytogenes* isolates (*Lm4*,* Lm33*, and* Lm28–31*) are consistent with results from previous studies on low-virulent* L. monocytogenes* field strains [10–12]. The mutations Ala117Thr and Val132Ile in InlB are located in the LRR domains of this protein, which are directly related to the interaction of this internalin with the Met cellular receptor and might compromise the adhesin function of InlB [11, 12]. The Ile17Val and Phe119Tyr mutations in PI-PLC are located in the signal sequence and the X-box domain, respectively, whereas the Thr262Ala mutation causes the introduction of an amino acid with different physicochemical properties, which might inhibit PI-PLC activity [12].

The mutations identified in PC-PLC, InlC, InlA, PrfA, and Hly are novel. The Ile13Thr mutation in PC-PLC is not located at the zinc-dependent phospholipase C domain of this protein, and the Ile10His and Met12Thr mutations in InlC are not located in the LRR domains of this internalin. The Thr51Ala and Ile157Leu mutations in InlA are also novel, and although they do not cause the truncation of InlA [11, 12], they are located in the LRR domains; therefore, these mutations might compromise the internalization of* L. monocytogenes* in epithelial cells. Further expression studies are required to confirm whether these mutations affect the expression and function of these virulence factors.

The low-hemolytic* L. monocytogenes* isolates contained the same Hly mutations as the atypical* L. innocua*; theconsequence of this observation is unclear. The deletion in* prfA *in the low-hemolytic* L. monocytogenes* isolates might underlie the reduced hemolytic activity in these strains because PrfA is the activator of the LIPI-1 cluster. However, the impairment of* prfA* would result in the reduced expression of all LIPI-1 genes. Therefore, further transcriptomic studies are required to completely characterize these atypical isolates, enhance our knowledge of their evolution and impact on public health, and develop more efficient methods for the identification and distinction of* Listeria* species.

## Figures and Tables

**Table 1:** Sources and phenotypic and genotypic characteristics of the *Listeria monocytogenes* isolates used in this study.

Isolate	Species	Serotype	Origin	Site	Year	ALOA	Hemolysis	*inlA *	*inlB *	*inlC *	inlJ*	*plcA *	*plcB *	*prfA *	*hly *
*Lm1 *	*L. monocytogenes *	1/2a	Slaught 1	Floor	2008	Halo	Positive	+	+	+	+	+	+	+	+
*Lm2 *	*L. monocytogenes *	1/2b	Slaught 1	Floor	2008	Halo	Positive	+	+	+	+	+	+	+	+
*Lm3 *	*L. monocytogenes *	4b	Market 1	Floor	2008	Halo	Positive	+	+	+	+	+	+	+	+
*Lm21 *	*L. monocytogenes *	1/2a	Slaught 1	Floor	2008	Halo	Positive	+	+	+	+	+	+	+	+
*Lm22 *	*L. monocytogenes *	1/2a	Slaught 1	Floor	2008	Halo	Positive	+	+	+	+	+	+	+	+
*Lm23 *	*L. monocytogenes *	1/2a	Slaught 1	Floor	2008	Halo	Positive	+	+	+	+	+	+	+	+
*Lm25 *	*L. monocytogenes *	1/2a	Slaught 1	Floor	2008	Halo	Positive	+	+	+	+	+	+	+	+
*Lm26 *	*L. monocytogenes *	1/2a	Slaught 1	Floor	2008	Halo	Positive	+	+	+	+	+	+	+	+
*Lm27 *	*L. monocytogenes *	1/2a	Slaught 1	Floor	2008	Halo	Positive	+	+	+	+	+	+	+	+
*Lm28 *	*L. monocytogenes *	1/2a	Market 2	Pork	2008	Halo	Weak positive	+	+	+	+	+	+	+	+
*Lm29 *	*L. monocytogenes *	1/2a	Market 2	Pork	2008	Halo	Weak positive	+	+	+	+	+	+	+	+
*Lm30 *	*L. monocytogenes *	1/2a	Market 2	Pork	2008	Halo	Weak positive	+	+	+	+	+	+	+	+
*Lm31 *	*L. monocytogenes *	1/2a	Market 2	Pork	2008	Halo	Weak positive	+	+	+	+	+	+	+	+
*Lm34 *	*L. monocytogenes *	1/2a	Human	Blood	1989	Halo	Strong positive	+	+	+	+	+	+	+	+
*Lm35 *	*L. monocytogenes *	4b	Human	Blood	2004	Halo	Strong positive	+	+	+	+	+	+	+	+
*Lm36 *	*L. monocytogenes *	4b	Human	Blood	1977	Halo	Strong positive	+	+	+	+	+	+	+	+
*Lm37 *	*L. monocytogenes *	4b	Human	CSF	1982	Halo	Strong positive	+	+	+	+	+	+	+	+
*Lm38 *	*L. monocytogenes *	1/2b	Human	CSF	1983	Halo	Strong positive	+	+	+	+	+	+	+	+
*Lm39 *	*L. monocytogenes *	1/2a	Human	Placenta	1978	Halo	Strong positive	+	+	+	+	+	+	+	+
*Lm39a *	*L. monocytogenes *	1/2a	Human	Placenta	1978	Halo	Positive	+	+	+	+	+	+	+	+
*Lm40 *	*L. monocytogenes *	1/2a	Human	Blood	1985	Halo	Strong positive	+	+	+	+	+	+	+	+
*Lm41 *	*L. monocytogenes *	4b	Human	CSF	1997	Halo	Strong positive	+	+	+	+	+	+	+	+
*Lm42 *	*L. monocytogenes *	4b	Human	CSF	1997	Halo	Positive	+	+	+	+	+	+	+	+
*Lm43 *	*L. monocytogenes *	1/2a	Human	CSF	1983	Halo	Positive	+	+	+	+	+	+	+	+
*Lm4 *	*L. monocytogenes *	1/2a	Market 2	Floor	2008	Halo	Weak positive	+	+	+	+	+	+	+	+
*Lm33 *	*L. monocytogenes *	1/2a	Market 2	Floor	2008	Halo	Weak positive	+	+	+	+	+	+	+	+
*Lm10 *	*L. monocytogenes *	4b	ATCC 19115	—	—	Halo	Strong positive	+	+	+	+	+	+	+	+
*Lm15 *	*L. monocytogenes *	1/2a	ATCC 19111	—	—	Halo	Strong positive	+	+	+	+	+	+	+	+

Slaught 1: slaughterhouse 1. CSF: cerebrospinal fluid. *All isolates were positive for fragments of *inlJ* but presented variable results for hole gene amplification (see Table 4).

**Table 2:** Sources and phenotypic and genotypic characteristics of the *Listeria innocua* isolates used in this study.

Isolate	Species	Serotype	Origin	Site	Year	ALOA	Hemolysis	*inlA* ^x^	*inlB *	*inlC *	*inlJ *	*plcA *	*plcB *	*prfA *	*hly* ^x^
*Lin5 *	*L. innocua *	6a	Market 1	Floor	2008	Halo*	Weak positive**	−	−	+	−	+	−	−	+
*Lin6 *	*L. innocua *	6a	Slaught 2	Floor	2008	Halo*	Weak positive**	−	−	+	−	+	−	−	+
*Lin7 *	*L. innocua *	6a	Slaught 2	Floor	2008	Halo*	Weak positive**	−	−	+	−	+	−	−	+
*Lin8 *	*L. innocua *	6a	Slaught 2	Floor	2008	Halo*	Weak positive**	−	−	+	−	+	−	−	+
*Lin9 *	*L. innocua *	6a	Slaught 2	Floor	2008	Halo*	Weak positive**	−	−	+	−	+	−	−	+
*Lin16 *	*L. innocua *	6a	Slaught 1	Floor	2006	Negative	Negative	−	−	−	−	−	−	−	−
*Lin17 *	*L. innocua *	6a	Slaught 1	Floor	2006	Negative	Negative	−	−	−	−	−	−	−	−
*Lin18 *	*L. innocua *	6a	Slaught 1	Floor	2006	Negative	Negative	−	−	−	−	−	−	−	−
*Lin19 *	*L. innocua *	6a	Slaught 1	Floor	2006	Negative	Negative	−	−	−	−	−	−	−	−
*Lin20 *	*L. innocua *	6a	Slaught 1	Floor	2006	Negative	Negative	−	−	−	−	−	−	−	−
*Lin11 *	*L. innocua *	6a	ATCC 33090	—	—	Negative	Negative	−	−	−	−	−	−	−	−
*Lin46 *	*L. innocua *	6a	CLIP 12612	—	—	Negative	Negative	−	−	−	−	−	−	−	−

Slaught 1: slaughterhouse 1; Slaught 2: slaughterhouse 2. *Subtle halo. ** Very weak positive hemolysis. ^x^Atypical isolates were positive for fragments of *inlAB* operon and *hly* but presented variable results for *inlA* and *hly* complete amplification (see Table 4).

**Table 3:** Primers designed in this study for the amplification of the *L. monocytogenes* virulence genes.

Primer	Sequence 5′-3′	Target	Product (bp)
*inlA ext Fw *	CGGCTCCGTAGACAGATTAG	*inlA *	2884
*inlA ext Rv *	GTGATAGTCTCCGCTTGTAC		
*inlA In*_ 1_ *-Fw *	GTGAGAAGAAAACGA		1200
*inlA Detec-Rv *	TGGTGTAAGATCGCT		
*inlA Detec-Fw *	AAGTGATATAACTCC		—

*inlB ext Fw *	GCTAGATGTGGTTTTCGGACT	*inlB *	2146
*inlB ext Rv *	TAAGCAGCGCAAAGGTGATTCCTAC		
*inlB In-Fw *	GTGAAAGAAAAGCAC		1227
*inlB Seq*_ 3_ *-Rv *	ATTCCCGCGAATATA		
*inlB Seq*_ 2_ *-Fw *	TGATGGAACGGTAAT		900
*inlB End*_ 3_ *-Rv *	TNATTTCTGTGCCCT		

*plcB ext Fw *	CCATACGACGTTAATTCTTGCAATG	*plcB *	1039
*plcB ext Rv *	TATCCACCCGCTAACGAGTG		

*plcA ext Fw *	GAGGTTGCTCGGAGATATAC	*plcA *	1100
*plcA ext Rv *	CTGCTGTCCCTTTATCGTCG		
*plcA Detec-Fw *	AACCATTATTATGCG		396
*plcA Detec-Rv *	TGCAGCATACTGACG		

*hly ext Fw *	CGATAAAGGGACAGCAGGACT	*hly *	1796
*hly ext Rv *	AGCCTGTTTCTACATTCTTCACAA		
*hly Detec-Fw *	TAACAACGCAGTAAA		566
*hly Detec-Rv *	CGTAAGTCTCCGAGG		
*hly End-Fw *	CCTCCTGCATATATC		725
*hly End-Rv *	TTATTCGATTGGATT		

*inlC In*_ 1_ *-Fw *	ATGCTAGTNTTAATTGTA	*inlC *	852
*inlC End*_ 2_ *-Rv *	CTAATTCTTGATAGGTTGTG		

*prfA Detec-Fw *	CTGCTAACAGCTGAGCTATG	*prfA *	404
*prfA Detec-Rv *	GCTACCGCATACGTTATC		
*prfA End Rv *	ATGAACGCTCAAGCA		—

In: primers corresponding to the beginning of the gene; End: primers corresponding to the end of the gene; Detec: internal primers designed for gene detection; ext: external primers; Seq: internal primers designed for sequencing.

**Table 4:** Distribution of the results of the virulence gene amplification from *Listeria* species.

Primer	Species	Positive	Negative
		*N* (%)	*N* (%)
*inlC_Liu* ^1^	*L. monocytogenes *	28 (100.0)	0
	*L. innocua *	0	12 (100.0)
*inlC In*–*End *	*L. monocytogenes *	28 (100.0)	0
	*L. innocua *	5 (41.7)	7 (58.3)
*prfA Johnson*–*End *	*L. monocytogenes *	28 (100.0)	0
	*L. innocua *	0	12 (100.0)
prfA_Johnson^2^	*L. monocytogenes *	28 (100.0)	0
	*L. innocua *	0	12 (100.0)
*prfA Detec *	*L. monocytogenes *	28 (100.0)	0
	*L. innocua *	0	12 (100.0)
*plcA ext *	*L. monocytogenes *	28 (100.0)	0
	*L. innocua *	5 (41.7)	7 (58.3)
*plcA Detec *	*L. monocytogenes *	28 (100.0)	0
	*L. innocua *	5 (41.7)	7 (58.3)
*plcB ext *	*L. monocytogenes *	28 (100.0)	0
	*L. innocua *	0	12 (100.0)
*inlB In*– *Seq*_3_	*L. monocytogenes *	28 (100.0)	0
	*L. innocua *	0	12 (100.0)
*inlB Seq*_2_	*L. monocytogenes *	28 (100.0)	0
	*L. innocua *	0	12 (100.0)
*inlA In*–*Detec *	*L. monocytogenes *	28 (100.0)	0
	*L. innocua *	0	12 (100.0)
*inlAB_Jung* ^3^	*L. monocytogenes *	28 (100.0)	0
	*L. innocua *	5 (41.7)	7 (58.3)
*hly ext *	*L. monocytogenes *	28 (100.0)	0
	*L. innocua *	0	12 (100.0)
*hly End *	*L. monocytogenes *	28 (100.0)	0
	*L. innocua *	5 (41.7)	7 (58.3)
*hly_Border* ^4^	*L. monocytogenes *	28 (100.0)	0
	*L. innocua *	5 (41.7)	7 (58.3)
*hly Detec *	*L. monocytogenes *	28 (100.0)	0
	*L. innocua *	5 (41.7)	7 (58.3)
*inlJ_Liu* ^1^	*L. monocytogenes *	28 (100.0)	0
	*L. innocua *	0	12 (100.0)
*inlJ ext *	*L. monocytogenes *	23 (82.1)	5 (17.9)
	*L. innocua *	0	12 (100.0)

^1^Primers described by Liu et al. [16]. ^2^Primers described by Johnson et al. [6]. ^3^Primers described by Jung et al. [17]. ^4^Primers described by Border et al. [22].
